# Visual perception and macular integrity in non-classical CLN2 disease

**DOI:** 10.1007/s00417-022-05662-1

**Published:** 2022-06-02

**Authors:** Yevgeniya Atiskova, Jan Wildner, Eva Wibbeler, Miriam Nickel, Martin Stephan Spitzer, Christoph Schwering, Angela Schulz, Simon Dulz

**Affiliations:** 1 Department of Ophthalmology, University Medical Center Hamburg-Eppendorf, Martinistraße 52, 20246 Hamburg, Germany; 2 Department of Pediatrics, University Medical Center Hamburg-Eppendorf, Hamburg, Germany

**Keywords:** Vision, Macula, Retina, CLN2 disease, NCL, Retinal degeneration, Eye

## Abstract

**Purpose:**

Patients with CLN2 suffer from epileptic seizures, rapid psychomotor decline and vision loss in early childhood. The aim of the study was to provide longitudinal ophthalmic data of patients with confirmed genetic mutation and non-classical disease course, marked by later onset, protracted progression and prolonged life span.

**Methods:**

Prospective, observational study to assess visual acuity, retinal features (Weil Cornell Ophthalmic Score), central retinal thickness (CRT) measured by optical coherence tomography and general disease progression (Hamburg CLN2 motor language score) in non-classical CLN2 patients.

**Results:**

All patients received intracerebroventricular enzyme replacement therapy with cerliponase alfa. Mean age at last follow-up was 12.4 years; mean follow-up time 2.6 years. All cases demonstrated a stable Hamburg motor language CLN2 Score and Weill Cornell LINCL Ophthalmic Severity Score. Visual function remained stable in 4/6 patients, 2/6 patients showed a decrease, 4/6 cases had a stable CRT and 2/6 showed a reduction of CRT. One patient showed a massive macular thinning and low vision. A correlation with a specific mutation or age could not be verified.

**Discussion:**

The presented longitudinal study characterizes the variable ocular involvement in non-classical CLN2 disease and contributes to the natural history description. The functional and morphologic data outline the necessity of regular ophthalmic examination.

**Summary statement:**

Ocular phenotyping and description of retinal degeneration in non-classical CLN2 disease.


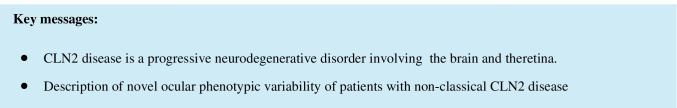


## Introduction

Neuronal ceroid lipofuscinoses (NCLs) form a heterogeneous group of neurodegenerative lysosomal storage disorders. To date, the group consists of 13 genetically different disease entities, which are classified by the underlying gene mutation (*CLN1-14*) [1]. All NCL diseases except one (CLN4) are autosomal recessively inherited. Their shared clinical hallmarks encompass psychomotor regression, epilepsy and vision loss resulting in premature death [2–4]. An internationally agreed NCL disease nomenclature is based on both the underlying genetic defect as well as the phenotype [5]. Most NCL diseases have a most prevalent “classic” phenotype. However, due to the increasing implementation of next generation sequencing panels and exome sequencing as essential diagnostic tools for the assessment of rare diseases, more and more patients are identified with “non-classical” phenotypes caused by mutational effect that lead to varying loss of function of the associated protein.

Neuronal ceroid lipofuscinosis type 2 (CLN2; OMIM 204,500) is caused by mutations in the *CLN2* gene resulting in deficient activity of the lysosomal enzyme tripeptidyl peptidase 1 (TPP1) [6]. Patients with the classic late infantile phenotype present between ages 2 and 4 years with seizures and language developmental delay, followed by rapid deterioration of motor, language and cognitive skills over a 2- to 3-year period and premature death [7, 8]. In patients with the classic late infantile CLN2 phenotype, seizures and psychomotor decline precede the vision loss [9]. Electroretinogram studies reveal subnormal or completely extinguished amplitudes as well as prolonged rod responses and severely subnormal cone b-wave responses [10]. Visual deterioration seems to occur after 3 years of age, although slight retinal abnormalities can be visible in presymptomatic patients in optical coherence tomography (OCT) imaging prior to obvious funduscopic changes. In OCT imaging, a bilateral symmetric progressive loss of central retinal thickness (CRT) could be shown especially between 4 and 6 years of age. The OCT-based documentation of the retinal degeneration correlated well with neurological scores and age in the classic late-infantile CLN2 disease [9].

Patients with non-classical CLN2 phenotypes are reported to have a disease later onset and a protracted progression of the disease. Most non-classical CLN2 affected patients show prolonged life span [11–17]. Some of the typical NCL symptoms such as epileptic seizures [18] or visual failure [14] can be absent or the order of symptom onset can vary. A systematic work assessing the retinal involvement in non-classical CLN2 patients has not been presented yet.

To date, CLN2 disease is the only NCL disease for which an approved pharmacological treatment exists. This treatment consists of intracerebroventricular enzyme replacement therapy (ICV-ERT) with Cerliponase alfa, the recombinant form of human TPP1 enzyme, which is deficient in these patients. This treatment has been shown to attenuate the loss of motor and language function in individuals with CLN2 disease [19]. However, this treatment primarily targets the central nervous system and not the retina. Additional therapies targeting vision loss in CLN2 patients are necessary, and first experimental therapy trials using approaches such as gene therapy are under development.

The aim of this study was to collect longitudinal data on the course of the ophthalmic phenotype in patients with non-classical CLN2 and to provide natural history data on the progression of visual and retinal degeneration for upcoming novel clinical trials.

## Methods

### Study design and participants

This is a prospective, observational study based on analysis of medical history data as well as pediatric, neurologic and ophthalmologic exams performed during standard of care routine consultation visits at the University Medical Center Hamburg-Eppendorf, NCL specialty clinic at the Department of Pediatrics and at the Department of Ophthalmology. These data were collected on patients who met the following criteria: (1) confirmed diagnosis of CLN2 disease based on deficient or reduced enzyme activity of tripeptidyl peptidase 1 (TPP1) and based on identification of a pathogenic mutation on both alleles of the *CLN2* gene, and (2) showed later symptom onset (> 5 years) and slower disease progression compared to the classic late infantile CLN2 phenotype. The study protocol of this observational study was approved by the local ethical committee of the Ärztekammer Hamburg (PV7215), and informed consent was obtained for all patients prior to enrollment.

### Procedures

Data on medical history prior to diagnosis and enrollment, first symptoms and diagnostic summary were collected for all patients in the DEM-CHILD database (clinicaltrials.gov identifier NCT04613089). Neurologic CLN2 disease progression was quantitatively assessed for each patient by pediatricians from the NCL-specialty clinic using a disease specific CLN2 Clinical Rating Scale [8], the Hamburg LINCL scale. The scale consists of four domains (motor, language, epilepsy, vision) and is scored from 0 to 3, whereby 3 points imply a normal domain function. The vision domain in particular is based on recognizing and grabbing a desirable object (3 points), grabbing for objects uncoordinated (2 points), the reaction to light ( 1 point) and no reaction to light stimuli (0 points). To improve the rating scale, the epilepsy and vision domain were excluded as both domains either altered by anti-epileptic treatment or on cognitive and motor function.

As part of their standard-of-care biannual ophthalmologic assessments, all patients underwent an anterior segment and dilated fundus exam and a swept source optical coherence tomography (SS-OCT) exam (Topcon DRI OCT Triton Plus, Topcon Medical Inc., Tokyo, Japan).

A macular cube (256 line scans) scan was performed in each eye, and central retinal thickness (CRT) measurements (µm) were extracted from the ImageNet 6 software (Topcon Medical Inc., Tokyo, Japan). As retinal degeneration is a highly symmetric process in CLN2 disease, both CRT values were averaged for further evaluation.

Depending on disease stage and severity of psychomotor impairment, best corrected visual acuity (BCVA) was tested by tumbling E hook test or a Lea symbol test. As monocular testing was partly not tolerated, binocular testing results were used for statistical analysis.

CLN2 associated funduscopic features (optic disc pallor, macular pigmentary changes, vascular attenuation and peripheral pigmentary changes) were examined, graded and scored by two independent ophthalmologists according to the Weill Cornell LINCL Ophthalmic Severity Score [20], a five severity clinical rating scale (a score of 1 representing no evidence of retinal degeneration, and an ophthalmic score of 5, exhibiting the most profound, end-stage retinal degeneration), and averaged as a symmetric score of both eyes was present for in each examination.

Except for descriptive statistics, no additional statistical analysis was performed due to the small sample size.

## Results

A total of six patients (4 females, 2 males) with genetically and enzymatically confirmed CLN2 disease and non-classical phenotype were included in this observational study. All patients had pathogenic mutations on both alleles of the *CLN2* gene. TPP1 enzyme activity measurements in dried blood spot samples showed very low residual enzyme activity of 0.01 nmol/spot*45 h in four patients and completely deficient enzyme activity in two patients. No obvious correlation could be found between the enzyme activity and the ocular phenotype (Table 1).

**Table 1:** Demographics and clinical features of non-classical CLN2 patients

Code	Gender	Genotype	TPP1 enzyme activity (nmol/spot*45 h) (range:0.1–1.2)	Age at first symptoms in years	Type of first symptom	Age at examination in years	Visual acuity	CRT average [µm]	Weill Cornell LINCL Ophthalmic Severity Score	Hamburg CLN2 Motor Language Score
A1	Female	c.509-1G > C; c.1333–1344dup	0	8.3	Ataxia, language regression	11.1	20/100	251	1	4
						11.5	20/32	256	1	4
						12.0	20/40	247	1	4
						12.4	No data	244	1	4
						13.0	20/40	238	1	4
						13.8	20/40	235	1	4
						14.3	20/40	233	1	4
						14.9	20/50	234	1	3
A2	Male	c.3G > C; c.1261 T > A	0	4	Language difficulties, dementia	9.9	No data	281	1	2
						10.3	20/40	275	1	2
						10.9	20/50	No data	1	2
						11.3	20/100	264	1	2
						11.8	20/100	267	1	2
						12.3	20/100	263	1	2
						13.1	20/100	264	1	2
						13.5	20/80	275	1	2
A3	Female	c.622C > T; c.1340G > A	0.01	6.3	Ataxia	12.4	20/500	120	4	3
						12.8	No data	118	4	3
						13.3	20/630	123	4	3
						13.8	20/500	125	4	3
						14.2	20/630	130	4	3
						14.6	20/1000	123	4	3
						15.1	No data	127	4	3
						15.5	20/630	125	4	3
A4	Female	c.509-1G > C; c.1439 T > G	No data	6	Learning difficulties	7.6	20/63	234	1	4
						7.7	20/32	No data	1	4
						8.2	20/63	No data	1	4
						8.7	20/63	238	1	4
A5	Female	c.509-1G > C; c.1627G > A	0	4.3	Seizures, language difficulties	5.8	20/50	201	2	4
						6.3	20/40	199	2	4
						6.8	No data	200	2	4
						7.2	No data	201	2	4
						7.8	20/50	197	2	4
A6	Male	c.622C > T; c.38 T > C	0.01	10	Ataxia, language regression	11.9	20/20	243	1	4
						12.3	20/20	239	1	4
						12.5	20/20	240	1	4
						13.0	20/20	238	1	4
						13.4	20/20	242	1	4
						13.9	20/20	237	1	4

All patients had an onset of first symptoms at age 4 years or older (range 4 to 10 years) which classified them as having a non-classical CLN2 phenotype. First symptoms were cognitive decline in two patients, a combination of ataxia and language regression in two patients, ataxia in one patient and epilepsy in one patient (Table 1).

The mean age at enrollment into this observational study was 9.8 years (SD 2.6 years, range 5.8–12.4 years). All patients started ICV-ERT with Cerliponase alfa at enrollment as approved therapy for the neurologic symptoms. A total of four patients had a Hamburg CLN2 motor language (ML) score of 4 at enrollment after having lost already one scoring unit each in the motor and language category each. Two patients had progressed further with ML scores of 3 and 2, respectively, at enrollment. The vision category in the Hamburg scale is not based on an ophthalmic exam, but a neurologic assessment of fine motor function such as coordinated versus uncoordinated grabbing for a desired object. At enrollment, five out of six patients had a full score of 3 in the vision category of the Hamburg scale, one patient (patient A1) had a score of 2 (data not shown).

The mean follow-up time for all patients in this study was 2.6 years (range: 1.1–3.8 years). Mean age at last follow-up was 12.4 years (SD 3.3 years, range 7.8–15.5 years). At last follow-up, the Hamburg ML score remained unchanged in five patients. One patient, who had been enrolled with an ML score of 4, lost one scoring unit in the motor domain (Table 1). The scores of the vision category in the Hamburg scale were unchanged in all six patients throughout the entire period of observation.

Descriptive statistical analysis of the ophthalmic exams showed a mean Weill Cornell LINCL Ophthalmic Severity Score at enrollment of 1.67 (range: 1–4; SD 1.2) and same values at last follow-up, respectively. A mean CRT of 221.4 μm (range: 123.0–280.9 μm) was recorded at enrollment, and a mean CRT of 212.75 μm (range: 124.5–274.5 μm) was recorded at last follow-up (Fig. 2). Visual acuity assessment was performed in five out of six patients with a Lea Symbols Distance visual acuity charts and in one patient (patient A6) with tumbling “E” eye chart examination. Visual acuity was normal (20/20) in 1 out of 6 patients and reduced (< 20/20) in 5 out of 6 patients at enrollment. In 2 out of 6 patients, a decline in visual acuity was observed during the study. However, fluctuations were noticed between examinations (Fig. 3).

Individual patients’ results from ophthalmic and neurologic assessments were as follows:

- Patient A1

The age at neurodegenerative symptom onset was 8 years, and these symptoms comprised ataxia and language regression with mild cognitive impairment. Patient had already suffered from mild language developmental delay at age 2 years. At 9.7 years, she had her first epileptic seizure and started showing first signs of vision loss. Patient enrolled in this observational prospective study at age 11.1 years with a Hamburg LINCL ML score of 4 just prior to start of ICV-ERT. The ML score remained stable at 4 until age 14.9 years when patient’s ataxia became worse with an ML score of 3. The first ophthalmic assessment was performed at the age of 11.1 years. Visual acuity was 20/100 at this first assessment, but 20/32 at the second visit after the examination was familiar. Follow-up assessment over the course of 3.8 years showed a decrease of visual acuity of 20/50. OCT imaging demonstrated an intact foveal and perifoveal outer retinal layer configuration over time and a mild decrease in CRT over time from initially 251 to 234 microns.

- Patient A2

The age at neurodegenerative symptom onset was 4.0 years with cognitive regression. At the age of 5 years, ataxia and language regression followed after patient had already suffered from mild language developmental delay at age 2 years. At 10 years, he had his first epileptic seizure. Patient enrolled in this observational prospective study at age 9.9 years with a Hamburg LINCL ML score of 2 just prior to start of ICV-ERT. The ML score remained stable at 2 throughout the follow-up time of 3.6 years. Visual acuity measurements declined mildly with an initial visual acuity of binocular 20/40 at age 10.3 years to 20/80 at age 13.5 years over at total of 3.2 years. However, OCT imaging presented an intact foveal configuration without signs of outer retinal layer degeneration in all 6-monthly examinations during this period and a CRT of 281–275 microns.

- Patient A3

**Fig. 1:**
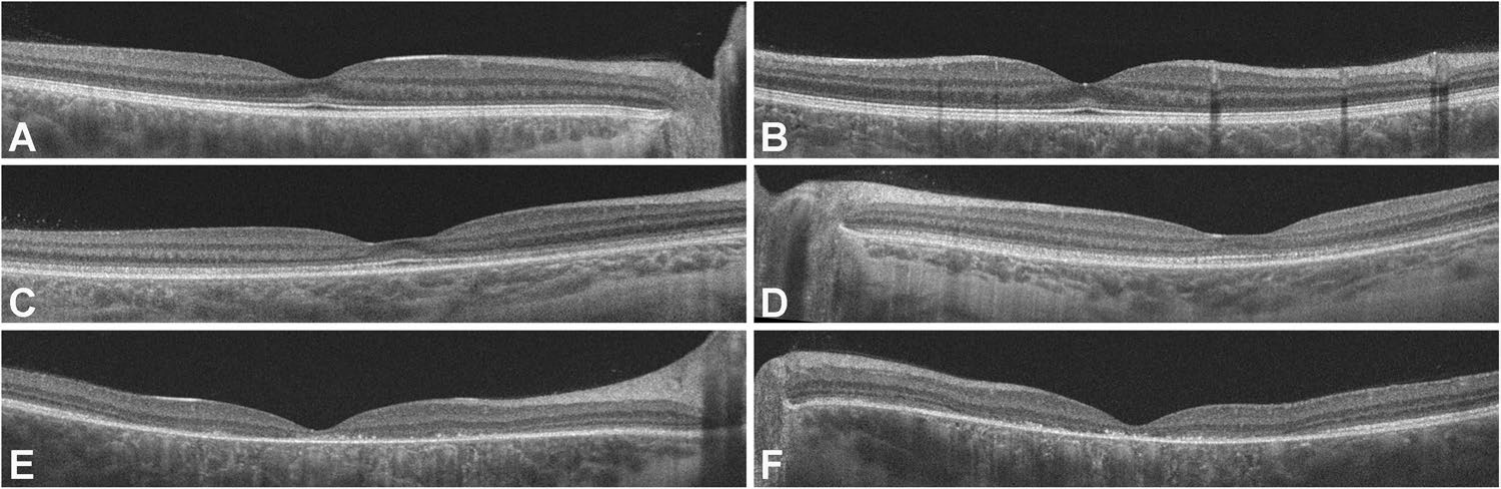
Regular macular configuration with preservation of the outer retinal layers and the ellipsoid zone in patient A6 (**A** right eye; **B** left eye) are displayed. In **C** (right eye) and **D** (left eye), a slight parafoveal thinning of the outer retina is visible (patient A5) on OCT imaging. In contrary, patient A3 (E right eye; F left eye) presents with vast outer retinal degeneration of the macula

The age at neurodegenerative symptom onset was 6.3 years with ataxia followed by cognitive and language decline at age 8.5 years and first seizures age 10.5 years. Patient enrolled in this observational prospective study at age 12.4 years with a Hamburg LINCL ML score of 3 just prior to start of ICV-ERT. The ML score remained stable at 3 throughout the follow-up time of 3.1 years. At the first ophthalmic assessment at the age of 12.4 years, binocular visual acuity on LEA symbols was documented with 20/500. At follow-up at the age of 15.5 years, a binocular vision was 20/630 with LEA symbols. OCT imaging demonstrated vast degeneration of the outer retinal layers within the macular region and a CRT of 120 microns (Fig. 1E/F), which remained stable over a period of 3.1 years. The Hamburg LINCL vision category score assessing the ability to grab for a desire object in coordinated way remained unchanged with a full score of 3 over the entire observation time.

- Patient A4

Neurodegenerative symptom onset was 6.0 years with mild cognitive decline followed by language regression at age 6.5 years and ataxia starting at age 7.5 years. Patient never had any seizures prior or during the observational study. Patient enrolled in this observational prospective study at age 7.6 years with a Hamburg LINCL ML score of 4 just prior to start of ICV-ERT. The ML score remained stable at 4 throughout the follow-up time of 1.1 years. First assessment of visual acuity at 7.6 years showed binocular 20/63 visual acuity on LEA symbols. Visual acuity remained unchanged over the entire follow-up period of 1.1 years. Similarly, a stable Weill Cornell LINCL Ophthalmic Severity Score of 1 and a stable CRT demonstrated no clinically relevant macular involvement in this patient.

- Patient A5

First symptom of CLN2 disease was epilepsy starting at age 4.3 years followed by ataxia age 5.6 years, and language regression at age 5.8 years. Patient enrolled in this observational prospective study at age 5.8 years with a Hamburg LINCL ML score of 4 just prior to start of ICV-ERT. The ML score remained stable at 4 throughout the follow-up time of 2.0 years. First assessment of visual acuity at age 5.8 years showed 20/50 binocular vision and remained stable until last follow-up at age 7.8 years. During this period, CRT remained stable and ranged between 201 and 197 microns. On OCT imaging at age 7.8, perifoveal RPE alteration and outer retinal layer thinning were observed (Fig. 1C,D).

- Patient A6

Neurodegenerative symptom onset was 10.0 years with ataxia and language regression. He enrolled in this observational prospective study at age 11.9 years with a Hamburg LINCL ML score of 4 just prior to start of ICV-ERT. The ML score remained stable at 4 throughout the follow-up time of 2.0 years. First assessment of visual acuity at 11.9 years of age showed binocular visual acuity with 20/20 on tumbling “E” hook exam and remained unchanged during 2.0 years of follow-up. CRT ranged between 243 and 237 microns over the period of follow-up (Fig. 1A,B). No macular involvement was documented on OCT at initial as well as all follow-up exams.

## Discussion

Late-infantile, classic and non-classical CLN2 disease phenotypes are differentiated based on age at clinical onset of specific neurological symptoms. Although retinal degeneration is one of the main characteristics of NCL diseases, only few data are published on ocular involvement in CLN2 disease. Classic late-infantile CLN2 disease is associated with rapid visual deterioration and macular degeneration in virtually all patients, while a detailed characterization of retinal features in non-classical CLN2 cases is missing. We first document an ophthalmic evaluation of CLN2 patients with a non-classical phenotype who have been followed longitudinally over numerous years. Our findings describe the peculiar parafoveal alterations (RPE alteration and/or outer retinal layer thinning) along with proposed options to evaluate visual function in non-classical CLN2 patients. All patients underwent serial longitudinal ophthalmological examinations over a period of 1.1–3.8 years capturing the course of ocular involvement, which represents a major benefit in contrast to one-time investigations especially in a non-classical disease course and contributes to the natural history description. Overall, ophthalmological data from patients from 5.8 years to 15.5 years of age are presented providing a wide spectrum of the disease course.

Even though affected by a milder, more slowly progressing disease course, all patients were in a progressive phase of the disease at enrollment and had lost scoring units in one or both categories for motor and language function (Table 1). Five of six patients demonstrated a stable Hamburg motor-language CLN2 score throughout the study period, which putatively might be due to successful treatment of their neurologic symptoms by ICV-ERT with Cerliponase alfa. However, natural history data on disease progression in untreated non-classical CLN2 patients are rare. All patients included had stable Weill Cornell LINCL Ophthalmic Severity Score values, which describes an established ophthalmic rating system for retinal features in CLN2 disease [20], suggesting no major changes in retinal morphology over the observational follow-up period.

The visual function remained stable in 4/6 patients, while 2/6 patients showed a decreased BCVA over the observational period; 2/6 patients suffered from a reduction of CRT over 10 µm, while all other cases had a stable CRT, measured by OCT imaging over the complete follow-up time.

**Fig. 2:**
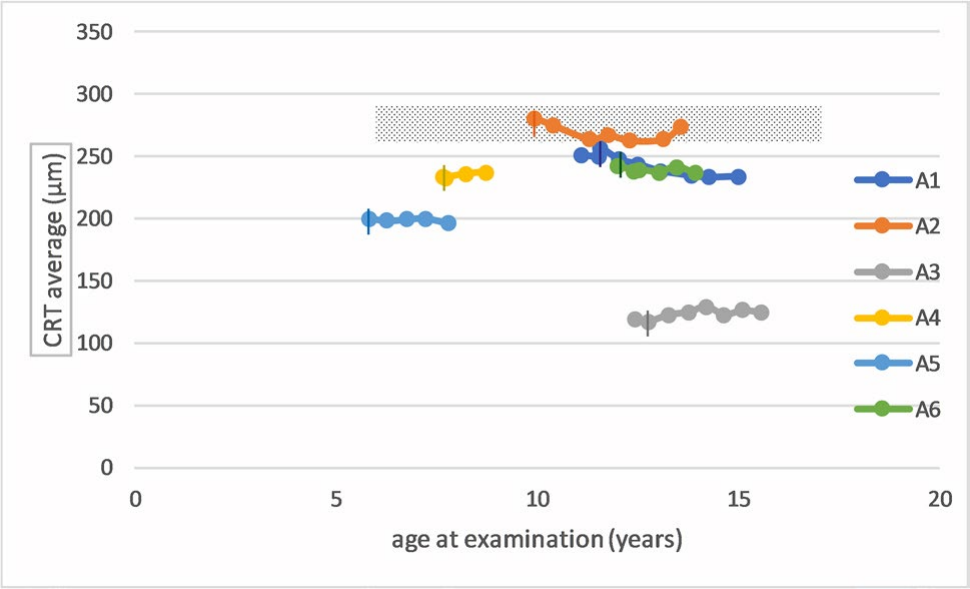
Central retinal thickness measurements over time assessed by optical coherence tomography. The grey box represents age adapted normal values (mean: 276 µm; standard deviation: 17.8 µm) according to Banc A et al. [21]

In total, 5/6 patients showed CRT values > 200 µm and BCVA values > 20/80, despite an advanced age of 7.8–14.9 at end of follow-up (Fig. 2.)

**Fig. 3:**
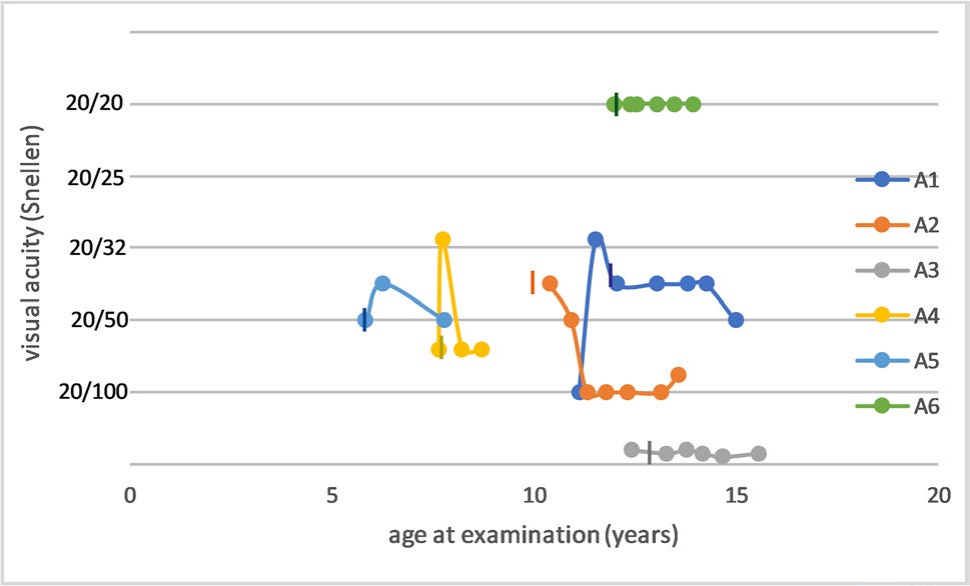
Longitudinal visual acuity measurements in non-classical CLN2 patients. A vertical line represents the start of intracerebroventricular treatment with cerliponase alfa

Only patient A3 showed a profound decrease in CRT (< 150 µm) and low BCVA (20/630) (Figs. 2 and 3). This particular case was followed up during the age of 12.4–15.5 years. The degenerated retinal status in this individual suggests a previous progressive outer retinal degeneration similar to classical affected CLN2 patients. Unfortunately, no ophthalmic examination data exist prior to enrollment. Previous examination by child neurologists—including the vision category of the Hamburg CLN2 score—did not reveal any vision problems and did not provide any additional information regarding the time point of the onset of retinal degeneration in this patient, as natural coping strategies masqueraded visual deterioration in earlier years. On the contrary, patient A1 achieved a score of 2 at the vision category, although measured visual acuity and CRT values were similar to patients A4 and A5. The vision category in the Hamburg CLN2 Score evaluates rather the fine motor function such as coordinated versus uncoordinated grabbing for a desired object than the visual function of the patients.

Kovacs et al. recently published the evolution of CLN2-associated retinal degeneration in classical affected patients. Their findings suggest that the retinal degeneration manifests as a progressive, symmetrical decline, appearing to accelerate during 48–72 months of age, suggesting intervention with retina-specific therapy occurs ideally before or as early as possible within this critical period [9]. Symmetric retinal thickness and macular configuration were found in non-classical CLN2 patients as well, yet in contrast to classic late-infantile CLN2 disease, retinal degeneration seems to affect not all patients with non-classical CLN2 disease.

Especially one case (A3) with good general condition but low visual function and low CRT values suggests a variable course of retinal degeneration in non-classical CLN2 patients. A correlation or dependence on a specific mutation, age, enzyme activity or other characteristics could not be verified by the presented study, due to a limited number of included individuals of the orphan disease. A consistent phenotype at a certain time point could not be observed. In order to identify potential clinical factors influencing the ocular disease course, upcoming clinical studies are essential. As our cohort of classical patients (28 patients; manuscript under review) does not show any benefit of intracerebroventricular treatment, we do not expect any treatment effect on the retinal status in non-classical CLN2 disease.

Although patients with non-classical CLN2 disease course may benefit from upcoming therapy approaches targeting the retinal degeneration, it is difficult to identify ocular affected patients and the age of onset of the retinal involvement. As a consequence of the latter case, we highly advocate routine ophthalmic visits in CLN2 patients to precisely document the ocular status. In contrast to classic late-infantile CLN2 patients, non-classical CLN2 patients are routinely capable to perform visual functions test and OCT imaging.
